# Transitional challenges faced by medical intern doctors (IDs) in Vanuatu: a qualitative study

**DOI:** 10.1080/10872981.2021.2005458

**Published:** 2021-11-25

**Authors:** Mackenzie Sitobata, Masoud Mohammadnezhad

**Affiliations:** a Dental Department, Norsup Hospital, Vanuatu Ministry of Health, Malekula, Vanuatu; b School of Public Health and Primary Care, Fiji National University, Suva, Fiji Islands

**Keywords:** Intern doctor, registered doctor, transition, challenges, Vanuatu

## Abstract

The transition from being a medical student to a fully qualified registered doctor is a challenging time in the lives of intern doctors (IDs). Throughout those challenging times they face many challenges which significantly impact their professional lives as well as their transitional experience. This study aimed to identify the transitional challenges experienced by IDs in Vanuatu. This qualitative study was conducted using a phenomenological approach whereby data collection is done through semi-structured in-depth interviews. Ethical approval was obtained before the commencement of this study. Twenty-seven participants were IDs of Vila Central Hospital and Northern Provincial Hospital in Vanuatu who were either current IDs and had worked for more than 6 months or had completed internship within the past 2 years. The willing IDs were consented on paper before they participated in the interview. The interview data was then transcribed verbatim and interpreted thematically. The participating IDs in the study were between the ages of 27 and 36 years old. Twenty two were current interns while the remaining five had recently completed their internship and now working as registered doctors. Three subthemes were identified as challenges through thematic analysis in this study; intern’s welfare not met; different medical training institution; and transitional shock. Those subthemes were later categorized. The study findings have identified that intern’s welfare needs improvement along with diverse training medical schools, and the transitional internship encounters were significant challenges experienced by IDs. There is indeed a need for healthcare providers, medical leaders, and relevant stakeholders to recognize and address these challenges.

## Introduction

The transitional journey of a graduate medical student into a qualified registered doctor is a remarkable journey that brings together different experiences that influence the professional life of an intern doctor (ID) [1]. IDs are new graduate medical students who have just began a career in medicine after attaining a medical qualification. Becoming a registered doctor it comes with increased responsibility and challenges which suddenly changes from a relatively protected environment to being expected to function competently on your own thus resulting in work challenges such as stress, distress, and burnout amongst new medical graduates [2]. Understandably, studying and practicing medicine is a physically, emotionally demanding career and training [3]. During internship doctors’ overall satisfaction levels are at their lowest in the first year after graduation from medical school and up to 19% of medical interns have some degree of psychological morbidity [4]. Overall, the purpose of the internship is to help new medical graduates to consolidate and apply their clinical knowledge, skills, and learn to take increasing responsibility for the provision of safe and effective patient care [5].

Most countries including Vanuatu have internship programs to support IDs in their transition. However, with the recent rise of returning medical students from different medical schools around the globe, a new medical internship program was reviewed and established to gather for such need in Vanuatu [6]. During medical internship which last for one to two years, interns must adapt to the new role, expectations, and deal with the emotions and challenges of professional integration which is intense and formative in the life of the IDs [7–9]. It is a kind of experiential learning during which recent graduates take the opportunity to apply acquired knowledge and skills from their medical school training to the real-world situations. In doing so it provides an opportunity for medical graduates to integrate and combine their thinking and actions [10–12]. Therefore, with the health human resource being a vital but scarce resource in Vanuatu, it needs to be well equipped to face the medical challenges out in the field with the right knowledge, skills, and qualities in order to perform with trust [13]. In third-world countries including Vanuatu, IDs encounter additional challenges including shortage of health sector budget, low income and health care disparities [14].

Medical internship program in Vanuatu has little history of how its internship program has started. However, it was understood that during the past years medical IDs in Vanuatu have been trained and supervised by expatriate medical expertise who have served in the country when no local expertise was produced. During 2016 the Vanuatu medical workforce team have reviewed the internship program for the first time as there is recent rise in numbers of returning graduate medical students in Vanuatu who graduated from different medical institutions around the globe [6].

The rise in returning numbers of foreign-trained medical graduates around the Western Pacific including Vanuatu were found to have varied in different level of clinical knowledge, skills, and foreign medical language used were challenging issues [15]. The Vanuatu Ministry of Health (MoH) under the internship and training committee have designed new internship program that will support IDs to develop confidence and competence in a supporting environment [16]. The findings from this study are relevantly important in developing appropriate strategies of support for the future IDs in such a way that will promote clinical skills development and creating a positive learning environment with positive experience. This study aims to close the gap by exploring the medical internship phase period as a transitional experience by the IDs and identify the challenges the interns faced during their internship transition period.

## Methodology

### Study design and setting

A qualitative study was used to gather information using face-to-face in-depth interviews from IDs in two referral hospitals in Vanuatu, namely Vila Central Hospital (VCH) and Northern Provincial Hospital (NPH). The two hospitals selected in this study were the only two hospitals in the country whereby the medical internship program was conducted since both referral hospitals have consultants and senior doctors to supervise and teach the interns. In-depth interviews was useful in this study as it uncovered in-depth details of interviewee’s experience and perspective on a subject [17,18].

### Study population and sample

All IDs in Vanuatu were considered as the study population in this study. Those who worked less than six months and were not willing to participate in the study were excluded. A convenient sampling method was best used in this study since IDs worked in shifts, rotations, and they may not be available at all times [19]. Therefore, convenient sampling was sorted from the research assistant by approaching the IDs during their working time, or after ward rounds or randomly coming across them in the hospital premises whereby arrangements for time and conducive location for the interview to be conducted was agreed on pending that the IDs are willing to participate in the interview. The face-to-face in-depth interviews were conducted until data saturation was reached. A total of 27 IDs participated in this study.

### Study procedures and tools

All participants were provided with an information sheet and signed written informed consent prior to conducting the face-to-face in-depth interview, using open ended questions. When using open-ended questions, it provides detailed and in-depth descriptions from an individual’s experience [20]. Open-ended questions were asked on the IDs first day experience and how they felt during their internship as a transition period from being a student to an intern doctor. Other discussions were to described the work challenges and the support that the IDs experienced with suggestions for improvements. A competent research assistant who had no affiliation with the participants was consented to conduct the interviews and participants were interviewed in their preferred local Bislama language. All responses were collected and kept confidential between the principal researcher and the research, including basic demographic data (gender, age, marital status). All interviewees were interviewed no more than an hour and were audio-taped for transcription by the researcher.

### Data management and analysis

A cross-translation was applied for translating the interviews from Bislama to English. All the interviews were transcribed by the researcher and was checked by the research assistant and a language translator to ensure that the transcription was transcribed accurately. The data were manually analyzed using thematic analysis process to identify the final themes. Thematic analysis is the method of analysis used in this study using the Braun and Clarke’s systemic model of thematic analysis [18] where it outlines what specific procedures and processes must be taken when doing a thematic analysis. The principle researcher read and reread each transcript line by line, identifying similar phrases and words than assigned numbers and colors to that word or concept. The coded data that had similar characteristics were grouped together. Once grouping of similar data was completed, descriptive themes and subthemes were identified to reflect the experiences described by the participants.

### Study rigor

Rigor in this study was observed according to the Lincoln and Guba strategies of credibility, dependability, confirmability and transferability [21]. Whereby in-depth interview questions as data collection tool were derived and formulated out from the previous academic sources and studies [22,23]. All participants were informed about the aim of study by telephone or email one week before conducting interviews. Purposive sampling was used to reach study participants. Consent was obtained from the research assistant before involving in the study. Details of the study and relevant information were provided to the study participants using an information sheet. The interviews were conducted at a time and venue that was suitable for both interviewer and interviewees. The process of study and conducting interviews, analysis data and emerging themes and sub-themes were discussed and feedbacks were provided by supervisor. An audit trial was provided, and researcher confirming as being a neutral person with no interference.

## Results

The demographical characteristics of the participants are provided in Table 1. A total of 27 participants were conducted for the data collection through in-depth interview. Out of the 27 participant 70% were male and 30% were female who were between the age of 27 tand 36. Twenty-two participants were still working as interns with remaining five had recently completed their internship no more than 2 years ago and were currently working in the two country’s referral hospitals. All participants were Ni Vanuatu, and 44% were in a de facto relationship, which means that the participants and their partners were in a relationship and live together as a couple but were not legally married.

**Table 1.** Participants demographic characteristics (n = 27)

Personal information	Frequency	Percentage
**Gender**		
Male	19	70
Female	8	30
**Age (Years)**		
27–31	11	40
32–36	16	60
**Ethnicity**		
Melanesia	27	100
**Marital Status**		
Single	11	41
Married	4	15
De facto relationship	12	44

Three themes were identified as; intern’s welfare not met; different medical training institution; and transitional shock, respectively with each two subthemes under the three themes (Table 2). In this section, participants are presented with a ‘P’ and cardinal number like P1, P2.

**Table 2.** Themes and subthemes identified from the interview

Theme	Subtheme
Intern’s welfare not met	- Accommodation. - Delay employment contracts and work without pay for a very long time.
Different medical training institution	- Language barrier - Different learning system and curriculum.
Transitional shock	- Poor orientation. - Assessed and work like registrar

## Theme 1: intern’s welfare not met

All [24,25] participants have expressed that during their internship they have gone through obvious challenges, and one of them is their concern for their welfare which was categorized as; the need for accommodation and the delaying employment contracts and work without pay for a very long time.

### Accommodation

Some participants [8] have raised their concern that they should be given accommodation near the hospital since they are working long hours and are taking first on calls. Another view in that matter to provide accommodation is that they have no financial means to fund their accommodation. P3, a 30-year-old female intern, noted:

*“so, we start having issues with our accommodation … inside those wards there are no sleeping rooms or bathrooms so you have to go home even if you are on call”*

Likewise, P6, a 32-year-old female intern, added that:

*“No resting space when you are on call. We use a building in the hospital compound but they deduct part of our salary. So, we have to leave and reside with families”*

### Delaying employment contracts and work without pay for a very long time

Every participant [24] stated that one of the greatest challenges they faced during their internship is the very long delay of their contract payments. Participants have expressed that they have gone without salaries for more nearly more than six months. P18, a 32-year-old male intern, stated that:

*“It is a very big issue we did not received wages for six to seven months … that’s our first-year internship so when our first-year contract ends then there is another delay for contract renewal into our second-year internship … a welfare issue we want it to be address, not continue”*

Similarly, another participant, P11, a 29-year-old male intern, claimed that delay of contract leads to delay of their internship.

*“One major issue we face when we came in was the process for approving our contract, when your contract is on hold you have to get a temporary leave and wait for your contract to be renew which takes two to three months than you have to come and start over again”*

## Theme 2: different medical training institution

All participants have studied in three different medical schools in three completely different countries; China, Cuba, and Fiji. A significant number of participants have experienced challenges during their internship in terms of language barrier and different learning system and curriculum.

### Language barrier

Twenty-one of the participants who attended medical universities in China and Cuba have admitted that one of the challenges experienced was language barrier on their return for internship. P4, a 32-year-old male intern, stated that:

*“I have to struggle with language barrier, since everything in Cuba is in Spanish. So, when we arrive in the medical ward, we have to translate this and that to Spanish to understand it”*

Similarly, P14, a 33-year-old female intern, added:

*“It’s a shock mainly with the language barrier where I graduated from everything was taught in China so the transition back was not easy”*

### Different learning system and curriculum

The twenty-one participants have also mentioned that the medical schools they attended in Cuba and China have different learning curriculum and learning system. Therefore, they have difficulties in terms of learning and clinical experiences. As, P12, a 32-year-old male intern, mentioned:

*“Our training back in Cuba was mostly theory, but for the practical side we lack it because plenty of us, and so according to the institution system, only the residents have the opportunity to do hands on”*

Similarly, P14, a 33-year-old female intern, who attended medical school in China shared her experience:

*“For me it takes five or six months. Because in China we hardly touch patient or we did not do practice that much, since it was strictly prohibited. We are more into theory”*

## Theme 3: transitional shock

All participants have described that they have experienced some form of transitional shock from their transitional change from being a student to being an intern medical doctor. Two of the transitional shocks were categorized as poor orientation and having interns being assessed and work like registrar.

### Poor orientation

Sixteen of the participants have noted that they had no proper orientation during the first early days of their internship program. P1, a 29-year-old female intern, recalls:

*“They told me which ward I will be working on, but they didn’t show me or introduce me to the department … after that I just walk around. They didn’t have a proper orientation”*

Likewise, P3, a 30-year-old female intern, described that:

*“There was no such thing as week of orientation to help us familiarized with the working environment”*

### Worked and assessed like registrars”

A few participants have noted that during their internship they were treated like registrars in terms of assigned tasks and assessing them. P2, a 28-year-old male intern, recalled that:

*“I know some of my class mates who are struggling where they are attached in, they are in shock because no more registrar with them”*

Another participant, P8, a 32-year-old male intern, stated that:

*“The worst is they evaluate us base on the question that is meant for registrar. These are difficult questions for interns”*

## Discussion

The present research to our knowledge is the first of its kind in Vanuatu as it describes the medical intern doctors’ numerous challenges they faced during the course of their internship period. The findings of this study substantiate the view of other studies which stated that intern doctors are faced with many challenges during their internship as a transitional phase into qualified registered doctors [22–26]. Three obvious challenges mentioned by the interns in this study are intern’s welfare not met, different medical training institution, and transitional shock.

As this study aims to identify the transitional challenges experienced by IDs in Vanuatu. Intern’s welfare not met was seen as the first challenge echoed by the interns whereby they have expressed the need for accommodation, delay employment contracts and work without pay. The findings of this study were convenient to the other studies whom have reported that lacking the right support and having poor infrastructure that is not conducive for the wellbeing of the IDs brings disturbance to their learning [23,26,27]. Likewise, other studies have stated that when there is poor support in; hospital management, medical intern doctor’s welfare, hospital staff, and academic opportunities, it greatly impacts an ID’s transitional phase from being a medical student to becoming a registered doctor [24,28]. Besides, other studies are indicative to say that hospitals as highly hierarchical working environment, medical interns have genuine limitations and difficulty in such cooperative organization whereby they cannot change the system they find themselves in [29–31]. Traditionally, Vanuatu being seen as a third-world country which relied heavily on donor partners. Suggestive additional challenges such as shortage of health sector budget, low income, and other disparities in health care distribution may have seen as contributing factors to the IDs welfare not being met [13,14]. However, similar studies have shown that intern’s satisfaction was influenced when there is improving of remuneration, working hours, and physical working conditions, while lack of orientation, lack of resources, and poor working environment influenced the internship experience [4,32].

The second inter related challenge mentioned by the IDs is the Different medical training institution. Since all participants in this study have studied in three different medical schools in three completely different countries: China, Cuba, and Fiji. The obvious encounters mentioned by the IDs during their internship is the language barrier and the different learning system and curriculum they have been thought in. It is quite challenging since the common language thought and learnt in by the two referral hospitals in Vanuatu were in English. Despite the context of rapid globalization, other studies have agreed to say that language speaking skills and communication initiatives were found to be critical factors in influencing the intern’s clinical experiences and competence [33,34]. Regardless of the challenge, the internship program in Vanuatu has provided what is known as pre-internship training program (PITP) to train and support the interns before they are capable enough to undertake the medical internship program [6]. Having this supporting mechanism in the internship program reduces stress and gives positive support to the IDs’ learning process [29,33].

Transitional shock is seen also as the other contributing challenge that the IDs have experienced in terms of poor orientation and having interns work and assessed like registrar. Those two unusual but interesting challenges identified in this study may have been suggestive of not enough medical registrars to assists and work beside the IDs, or it could be that consultants felt that the cap between the registrars and the consultants can be filled by the interns. This situation may have a great demoralizing impact in the lives of the IDs during their internship. Studies have shown that despite curriculum reform this transitional change can be very stressful as newly gained responsibility, managing uncertainty, change of support infrastructure, reduced clinical support and social withdrawal were seen as contributors to this shock [35–37]. However, notwithstanding such a view, it appears that other studies have shown that internship orientation program lays a strong foundation for naive interns and it reduces anxiety while increasing confidence and improving performance [38–40]. This have strongly suggested that at the early stages of the IDs internship it is crucial and seen to be the foundation. Conversely, medical students have to understand that transiting into being an intern, responsibilities are expected to increase in various settings and situation [26,41]. In the developed countries research have pointed out that a good introduction with an enriched orientation curriculum durably augments intern confidence reduces anxiety and stress which makes internship transition easy [35–44].

## Limitations

The logistic limitations of the study in terms of conducting the interview or reaching the study participants due to COVID-19 pandemic. Due to time constraints and the geographic archipelago settings of the country it was difficult to captured other medical interns who are out doing their rural block rotation.

## Conclusion

Three challenges were being identified in this study as; intern’s welfare not met; different medical training institution; and transitional shock as challenging experience faced by the medical intern doctors in Vanuatu. It is viewed that medical students need to prepare for the real demands of being a qualified doctor and the aligning challenges of transitioning to their new role as interns. Medical consultants, senior doctors and nurses must provide the right kind of learning and supporting environment that will reduce these challenges which will enhance a positive influence during the internship transition. Such working and learning environment contribute to the development and satisfaction of the intern doctors in terms of increasing their competence and confidence professionally. There is an obvious need for internship coordinators, supervisors and health administrators to fully commit and support the intern doctors in allowing them to grow and develop so that they can be able to provide the professional health care that is needed.
